# Progress of cereal transformation technology mediated by *Agrobacterium tumefaciens*

**DOI:** 10.3389/fpls.2014.00628

**Published:** 2014-11-07

**Authors:** Yukoh Hiei, Yuji Ishida, Toshihiko Komari

**Affiliations:** Plant Innovation Center, Japan Tobacco Inc., IwataJapan

**Keywords:** *Agrobacterium tumefaciens*, transformation, cereal, monocotyledon, gene transfer

## Abstract

Monocotyledonous plants were believed to be not transformable by the soil bacterium *Agrobacterium tumefaciens* until two decades ago, although convenient protocols for infection of leaf disks and subsequent regeneration of transgenic plants had been well established in a number of dicotyledonous species by then. This belief was reinforced by the fact that monocotyledons are mostly outside the host range of crown gall disease caused by the bacterium and by the failures in trials in monocotyledons to mimic the transformation protocols for dicotyledons. However, a key reason for the failure could have been the lack of active cell divisions at the wound sites in monocotyledons. The complexity and narrow optimal windows of critical factors, such as genotypes of plants, conditions of the plants from which explants are prepared, tissue culture methods and culture media, pre-treatments of explants, strains of *A. tumefaciens*, inducers of virulence genes, transformation vectors, selection marker genes and selective agents, kept technical hurdles high. Eventually it was demonstrated that rice and maize could be transformed by co-cultivating cells of callus cultures or immature embryos, which are actively dividing or about to divide, with *A. tumefaciens*. Subsequently, these initial difficulties were resolved one by one by many research groups, and the major cereals are now transformed quite efficiently. As many as 15 independent transgenic events may be regenerated from a single piece of immature embryo of rice. Maize transformation protocols are well established, and almost all transgenic events deregulated for commercialization after 2003 were generated by *Agrobacterium*-mediated transformation. Wheat, barley, and sorghum are also among those plants that can be efficiently transformed by *A. tumefaciens.*

## INTRODUCTION

**FIGURE 1:**
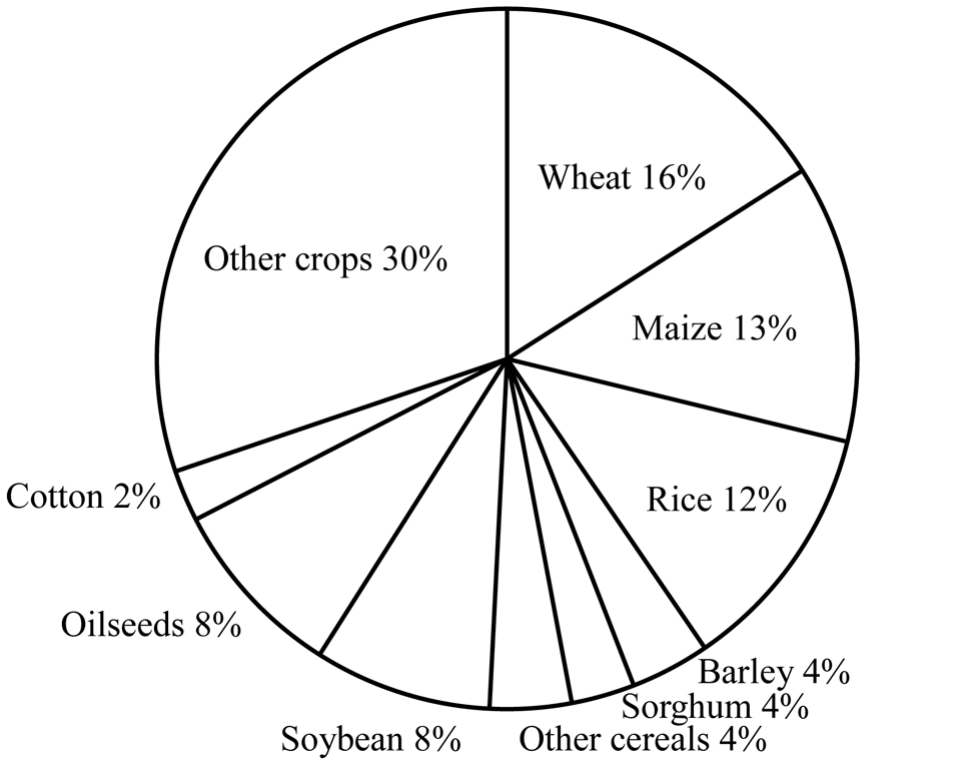
**Breakdown by crop of the arable land in the world based on the data from USDA World Agricultural Production 2014 http://apps.fas.usda.gov/psdonline/circulars/production.pdf**.

Whenever issues of global supply of food are discussed, a key subject is cereal grains, which are indeed staples for human beings. 2.5 billion metric tons of cereal grains per year are currently produced from about a half of the total arable land in the world (**Figure 1**; USDA World Agricultural Production 2014^1^). A serious issue is that the demand/supply balance of cereal grains is threatened by many factors. Human population on the planet Earth, which is currently 7.2 billion, is projected to reach nine billion by the middle of the 21st century (United Nations World Population Prospects^2^). This forecast could simply imply 30% more food will be needed in the next 40 years. Economic growth of developing countries will naturally create a demand for more meat, which will automatically require much more grain. Yet, no significant increase in cultivation area is expected during this period. Indeed, the tendency is the other way around; the world is losing farm land due to soil erosion and other environmental problems. Now, global warming could endanger even the current level of agricultural capacity (Intergovernmental Panel on Climate Change^3^).

There is no question that every means must be employed to sustain and elevate grain production. Biotechnology must be an essential component of the efforts. In every step of application of biotechnology to crop improvement, from basic studies to development of commercial varieties, gene transfer technology plays a key role. Dissection of biological processes at the molecular level in plants, examination of the gene effects, proper regulation of transgenes, and robust generation of transgenic events for commercialization in a crop of interest could all be effectively conducted if given genotypes of germplasm in the crop species can be transformed at a high frequency.

In dicotyledonous species, the gene transfer method of choice since the very early stage has been transformation mediated by the soil bacterium *Agrobacterium tumefaciens*. *A. tumefaciens* can transfer bacterial DNA (T-DNA or transfer DNA) to plant cells to incite plant tumors called crown galls (Ream, 1989). By the middle of the 1980s, the basic mechanism of tumorigenesis was elucidated, and the methods, in which explants such as leaf segments were co-cultivated with *A. tumefaciens*, to generate transgenic plants without inciting tumors were developed for tobacco, petunia, and other dicotyledons (Fraley et al., 1986). *A. tumefaciens* can transfer relatively large DNA segments with defined ends to plant cells with few rearrangements (Hooykaas and Schilperoort, 1992).

However, until the middle of the 1990s, efficient transformation of monocotyledons by *A. tumefaciens* was a fantasy. It was generally believed that *A. tumefaciens* could not transform monocotyledons because these plants are outside the host range of crown gall disease (De Cleene and De Ley, 1976). Then, the paradigm totally changed. Major cereal crops, rice (*Oryza sativa* L.), maize (*Zea mays* L.), wheat (*Triticum aestivum* L.), barley (*Hordeum vulgare* L.), and sorghum (*Sorghum bicolor* L.), are now efficiently transformed by *A. tumefaciens*.

In this review, we first outline studies of the early attempts to inoculate monocotyledons with *A. tumefaciens* and to establish efficient transformation methods. Then, we discuss progress and the current level of the gene transfer technology in major cereal crops, and both the importance across species of the key technology pieces and the advancement of the transformation protocols in each crop are summarized. The focus of the present article is literally on “gene transfer methods”; issues such as patterns of foreign DNA integration, transgene expression, gene targeting, and genome editing are reviewed extensively by other authors in this Research Topic.

## EARLY ATTEMPTS TO TRANSFORM MONOCOTYLEDONS

In spite of the general belief that *A. tumefaciens* could not transform monocotyledonous plants, many scientists had hoped that this hurdle could somehow be overcome. Quite a few laboratories studied interactions between monocotyledonous species and *A. tumefaciens* in one way or another. Simple tumor induction assays, such as one in *Asparagus* (Hernalsteens et al., 1984), and detection of the activity of the enzymes involved in the metabolism specific to tumor cells were conducted in a number of species including maize (Graves and Goldman, 1986). Douglas et al. (1985) and other groups found that *A. tumefaciens* could attach specifically to the cells of bamboo and other monocotyledons in the same manner as the bacterial cells attach to dicotyledonous plants. Although production of the compounds that induce the virulence genes, which are involved in the transfer of T-DNA, or the extent of induction by monocotyledonous plants were limited (Usami et al., 1987), identification of potent inducers, such as acetosyringone (Stachel et al., 1985), offered an option to induce the virulence genes by artificially added chemicals. Meanwhile, viral genomes were introduced into maize (Grimsley et al., 1987) and other cereals by *A. tumefaciens* to cause systemic infection. Expression of chimeric maker genes in cereals was then demonstrated, and kanamycin- or G418-resitant cells that expressed the gene for β-glucuronidase (GUS) were obtained in rice (Raineri et al., 1990), maize (Gould et al., 1991) and wheat (Mooney et al., 1991).

Breakdown of the steps involved in various transformation systems mediated by *A. tumefaciens* is summarized in **Table 1**. The studies mentioned above indicated that many of the steps were active in the interaction of cereals with *A. tumefaciens* and that DNA could be transferred from *A. tumefaciens* to cereal cells. As some of the steps in cereal transformation may be quite different from the ones of tumorigenesis, it might have been irrelevant in the first place to state that *A. tumefaciens* cannot transform monocotyledons for the reason that these plants are outside the host range of crown gall disease. On the other hand, critical reviews were published stating that some of the results were controversial and no firm proof of integrative transformation was presented (Potrykus, 1990).

**Table 1:** Steps in plant transformation systems.

Step	Tumorigenesis	Leaf segment transformation in dicotyledons	Cereal transformation	Floral transformation in *Arabidopsis*
Conditioning of plant cells	Cell division in wound response	Cell division in wound response	Cell division by tissue culture or young immature embryo cells	Ovules are transformation-competent.
Access to host tissues by bacteria	Thorough wounds close to the ground by strains naturally occurring in soil	Thorough wounds of explants by strains engineered with modified T-DNA during co-cultivation	Co-cultivation of plant tissues and strains engineered with modified T-DNA	Strains engineered with modified T-DNA could enter hosts through wounds and reach locules.
Association of bacteria with plant cells	Specific attachment by bacteria to plant cells	Specific attachment by bacteria to plant cells	Specific attachment by bacteria to plant cells	Specific attachment by bacteria to plant cells
Induction of virulence genes	By substances produced by wound response	By substances produced by wound response	By substances externally added	By substances naturally occurring in plants
T-DNA transfer	By virulence genes of bacteria	By virulence genes of bacteria	By virulence genes of bacteria	By virulence genes of bacteria
Integration of T-DNA to host chromosome	Mainly by host genes for DNA repair/synthesis	Mainly by host genes for DNA repair/synthesis	Mainly by host genes for DNA repair/synthesis	Mainly by host genes for DNA repair/synthesis
Cell division of transformed cells	Promoted by phytohormones produced by T-DNA genes	Supported by tissue culture	Supported by tissue culture	Natural development
Selective proliferation transformed cells	Uncontrolled cell growth	Selection marker and selective agent	Selection marker and selective agent	None
Destiny of infecting bacteria	Nourished by opines in tumor	Removed by antibiotics	Removed by antibiotics	No positive/negative pressure
Destiny of transformed cells	Tumors	Transgenic plants and progeny	Transgenic plants and progeny	Give rise to seeds by chance

## DEVELOPMENT OF EFFICIENT METHODS FOR CEREAL TRANSFORMATION

The controversy was eventually resolved, and efficient generation of transgenic cereals was reported in the mid 1990s. Transgenic plants were obtained from calli induced from mature embryos of rice (Hiei et al., 1994) and from immature embryos of maize (Ishida et al., 1996) after the tissues were co-cultivated with *A. tumefaciens*. The frequency of independent transformants obtained per infected explant was remarkably high, between 10 and 30%, and the results of Southern hybridization, Mendelian segregation of the T-DNA, and the analysis of the junctions between the T-DNA and plant DNA clearly demonstrated the stable integration and inheritance of T-DNA in these studies.

Potrykus (1990) commented that cereals are difficult to transform not because they are monocotyledons, but because they show no wound response. In a way, Hiei et al. (1994) and Ishida et al. (1996) supported this account by showing that the use of the cells which were actively dividing or about to divide and capable of regenerating plants was essential. The fact that cereal leaf segments were not good for transformation because they lacked wound responses is probably a reason why it was difficult initially to develop efficient leaf transformation protocols. This discussion is consistent with the observation that “conditioning of plant cells,” namely induction of cell division by wound responses, is a prerequisite for transformation (Kahl, 1982). In addition, unless meristematic cell lineage of transformed cells is generated by cell divisions, transformation will not produce sustained proliferation of cell mass. The choice of immature tissues and tissue culture technology supplied cells, which were actively dividing or about to divide and capable of regenerating plants, are critical for cereal transformation.

Hiei et al. (1994) and Ishida et al. (1996) also pointed out that numerous factors, types and stages of the tissues infected, the concentration of the inocula, tissue culture media, the type of the vectors, the strains of *A. tumefaciens*, the selection markers and selective agents, and the genotype of plants, were of critical importance. It is understandable that many factors were critically important because so many steps are involved (**Table 1**). This multiplicity of factors is another reason why transformation methods mediated by *A. tumefaciens* were not readily developed for cereals.

## STRAINS OF *A. tumefaciens* AND VECTORS FOR CEREAL TRANSFORMATION

The importance of the choice of the strains, vectors and markers is emphasized above. Yet, most of these components were not ones specifically designed for cereal transformation. The strains of *A. tumefaciens* co-cultivated with cereal cells were the strains previously employed for transformation of dicotyledons. Actually, only a limited number of strains have been used in most of the studies. One is strain LBA4404 (Hoekema et al., 1983), and another is a group of strains, such as EHA101, EHA105, AGL0, and AGL1, derived from strain A281, whose host range is wider and transformation efficiency is higher than other strains (Komari, 1989). These are the strains popular for transformation of dicotyledons. Transformation vectors introduced into these strains were again essentially the same vectors that were employed for dicotyledons. The superior capability of A281 was exploited in another way to enhance the competency of *A. tumefaciens* to transform plants. A segment from the virulence region of A281 was integrated into a transformation vector to generate a “super-binary vector” (Komari et al., 1996), which was introduced into LBA4404 and tested in tomato (Saito et al., 1992) before experiments were conducted in cereals.

*Agrobacterium* strains and vectors that take advantage of the capabilities of strain A281 have played important roles in cereal transformation. Maize was efficiently transformed with LBA4404 containing super-binary vectors (Ishida et al., 2007). *A. tumefaciens* strains LBA4404 and EHA101 were better than other strains in early rice transformation studies (Hiei et al., 1994). EHA101 and EHA105 were also the strains of choice for transforming wheat (Ishida et al., 2014). AGL1 was higher than LBA4404 in the frequency of transformation of barley (Hensel et al., 2008) and of sorghum (Wu et al., 2014).

Recently, a strain of *A. tumefaciens* that expressed 1-aminocyclopropane-1-carboxylic acid (ACC) deaminase, which could reduce ethylene production by plant cells, improved the delivery of T-DNA to *Erianthus ravennae* (Someya et al., 2013). Such a strain may improve the efficiency of transformation of various cereals.

## MARKER GENES FOR CEREAL TRANSFORMATION

Selection marker genes and visual expression marker genes for cereals were also the ones employed in dicotyledons before use in cereals. For rice transformation, a gene for hygromycin phosphotransferase (HPT) was the most efficient selection marker (Hiei and Komari, 2008). Genes for phosphomannose-isomerase (PMI), neomycin phosphotransferase (NPTII), and phosphinothricin acetyl transferase (PAT or BAR) were useful in rice and other cereals, but the preference depended on the species. Genes for PAT/BAR were the best marker for maize (Ishida et al., 2007) and wheat (Ishida et al., 2014). The HPT gene was chosen in barley (Hensel et al., 2008), and the PMI gene was preferred in sorghum (Wu et al., 2014).

Many types of visual expression marker genes, such as a gene for a fluorescent protein, are now available for virtually any plant species, but a GUS gene, especially the one that carried an intron in the coding sequence (Ohta et al., 1990), played an important role in the early studies of cereal transformation. Because this gene was not expressed in bacteria, the expression in cereal cells was clearly examined. Detection of the expression of visual maker genes after the co-cultivation of plant cells and *A. tumefaciens* is a very convenient method to monitor transformation.

An element specifically important for cereals was the presence of introns in the marker genes, which considerably enhanced gene expression in cereals (Ueki et al., 2004). The specificity and strength of a promoter are different in dicotyledons and in monocotyledons. The promoters of a maize ubiquitin gene (Toki et al., 1992) and a rice actin gene (Zhang et al., 1991) have been extensively employed in cereals.

## TISSUE CULTURE MEDIA FOR CEREAL TRANSFORMATION

A cereal transformation protocol typically consists of a series of tissue culture steps, such as co-cultivation of target tissues and *A. tumefaciens*, resting culture, selection culture for specific proliferation of transformed cells, regeneration culture and rooting culture. Tissue culture conditions for non-transgenic cells to support proliferation of dedifferentiated cells and regeneration into plants are prerequisites for efficient transformation protocols, and the media compositions for non-transgenic cells are good starting points for optimization of media for transformation protocols. Naturally, many of the media employed in cereal transformation protocols are derivatives of traditional media compositions, such as MS (Murashige and Skoog, 1962) and N6 (Chu, 1978). It is apparent that media compositions that have been tested in rice and maize are much more diverse and advanced than the ones in other cereals. For example, the media tested for wheat, barley, and sorghum so far have been only derivatives of MS media (Hensel et al., 2008; Ishida et al., 2014; Wu et al., 2014).

However, use of the media for the culture of non-transgenic cells may not be good enough, partly because cells must endure the stresses specific to the transformation process. Contact with *A. tumefaciens* is often a severe stress to plant cells. Selection pressure is also a severe abiotic stress. Even the transformed cells expressing a selection marker gene tend to grow less vigorously on the media that contain a selective agent than on non-selective media. Thus, more extensive optimization of culture conditions is needed both for alleviation of stresses and for general improvement of growth conditions of the cells. A number of factors are considered, and the types and concentrations of phytohormones, especially auxin, are often the first item on the list. 2,4-dichlorophenoxy acetic acid (2,4-D) is the most popular auxin for culturing cereals. Other chemicals, such as 3,6-dichloro-2-methoxybenzoic acid (Dicamba) and 4-amino-3,5,6-trichloro-2-pyridinecarboxylic acid (Picloram), were effective in some protocols, but indole-3-acetic acid (IAA) and 1-naphthaleneacetic acid (NAA) are not popular for cereal culture. In general, the level of auxin is higher in the earlier steps to promote undifferentiated cell divisions and lower in the steps of regeneration and rooting. Fine tuning of hormone compositions have been attempted on top of this basic rule. The types and concentrations of sugars are another target of modification. The osmotic pressure of the medium may be adjusted by sugars, and sometimes the replacement of one sugar with another, for example, sucrose with maltose, makes a big difference (Kuhlmann and Foroughi-Wehr, 1989; Biswas and Zapata, 1993). Mineral ions, such as Cu^2+^ and Ag^+^, have occasionally elevated the efficiency of transformation effectively in cereals (Ishida et al., 2003; Bartlett et al., 2008). Addition of specific amino acids or various mixtures of amino acids has also been tested. Certain modifications are conducted somewhat rationally. For example, addition of Ag^+^ is thought to make plant cells insensitive to ethylene produced by stressed cells (Beyer, 1976). However, modifications are made mostly by trial and error; key references are given below for each crop.

How the bacteria are removed after the infection is a key concern in transformation protocols. For this purpose, antibiotics such as cefotaxime, carbenicillin, and timentin are added to the media after co-cultivation of plant cells and *A. tumefaciens*. This process is sometimes not straightforward. Cefotaxime is potent for killing *Agrobacterium*, but it was also reported to inhibit the growth of cells in certain plant species (Nauerby et al., 1997). Carbenicillin may be a good alternative (Ishida et al., 2003), but some *Agrobacterium* vectors or strains carry carbenicillin resistance genes. Timentin is popular in the studies of wheat and barley (Tingay et al., 1997; Jones et al., 2005). Occasionally, bacteria overgrow during the co-cultivation period and are not easily removed. Washing plant cells with liquid media may help reduce the bacteria. Decreasing the amount of sugar and other components such as amino acids could slow bacterial growth without hindering the viability of plant cells. Lowering the temperature during the co-cultivation is another option. Although plant cell growth could also become less vigorous, in addition to the slower growth of bacteria, the T-DNA transfer machinery within the bacteria may be more active at a lower temperature (Fullner and Nester, 1996).

## PROGRESS IN RICE TRANSFORMATION

Rice may be considered as the leading crop in cereal gene transfer technology as numerous papers, 100s per year, have been reporting experiments using *Agrobacterium*-mediated transformation of rice for basic and applied studies. Rice is the first cereal crop species for which an efficient transformation protocol mediated by *A. tumefaciens* was developed (Hiei et al., 1994). Since then, extensive studies have achieved improvements in frequency of transformation and expansion of the range of transformable genotypes.

In the original protocol of Hiei et al. (1994), transgenic plants were obtained from calli induced from the scutella of mature seeds. Mature seeds are convenient materials because they can be stored on a laboratory shelf at room temperature for a very long time before transformation experiments are conducted, and good calli may be induced so long as the seeds can germinate. However, it may not be straightforward to induce calli useful for transformation except for a limited number of genotypes. Rice can be classified into two subspecies, indica and japonica, and indica is generally more recalcitrant to tissue culture and transformation. There are variations even within the subspecies in tissue culture responses. Transformation of calli is efficient still only for some japonica varieties and for a very limited number of indica cultivars.

Later studies revealed that the infection of immature embryos produced transformants much more efficiently than the infection of calli of diverse genotypes of both indica and japonica varieties (Hiei and Komari, 2008). Pre-treatment of the immature embryos with heat and centrifugal force further elevated transformation efficiency (Hiei et al., 2006) although the mechanisms behind the effects are not understood. The protocols were well optimized for immature embryos of many genotypes (Hiei and Komari, 2008). As many as 15 independent transgenic plants were obtained from a single immature embryo that was co-cultivated with *A. tumefaciens* from leading indica cultivars of the world, such as IR64 and IR72 (**Table 2**). Hiei and Komari (2008) predicted that there is a good chance that every genotype of rice can be transformed efficiently if immature embryos are used. However, they also commented that successful transformation depends on the quality of the embryos and that good embryos must be obtained from healthy plants, which are at the right developmental stage and growing vigorously in a well-conditioned greenhouse. They added that the size of the embryos is a good indicator of the stage and that embryos between 1.3 and 1.8 mm in length along the axis are good for transformation, although the time required for embryos to reach the best stage differs depending on the genotype and the season. Hiei et al. (1994) initially claimed that immature embryos were poor materials probably because they did not use immature embryos from plants grown under optimal conditions. Thus, immature embryos are potentially very good but sensitive and expensive tissues. A considerable investment is needed to set up a good greenhouse facility and to maintain a team of experienced greenhouse technicians and tissue culture scientists.

**Table 2:** Frequency of transformation in cereals and grasses mediated by *Agrobacterium tumefaciens*.

Species, Genotype	Target explant	Independent events explant	Reference
Rice (*Oryza sativa* L.), japonica (eg. Nipponbare, Koshihikari)	Immature embryo	18.0*	Hiei and Komari (2008)
Rice (*Oryza sativa* L.), indica (eg. IR64, IR72)	Immature embryo	15.0*	Hiei and Komari (2008)
Rice (*Oryza sativa* L.), japonica (eg. Nipponbare, Koshihikari)	Callus	0.9	Hiei and Komari (2008)
Maize (*Zea mays* L.), A188	Immature embryo	0.5	Ishida et al. (2007)
Wheat (*Triticum aestivum* L.), Fielder	Immature embryo	0.9	Ishida et al. (2014)
Barley (*Hordeum vulgare* L.), Golden Promise	Immature embryo	0.87	Hensel et al. (2008)
Sorghum (*Sorghum bicolor* L.), Tx430	Immature embryo	0.33	Wu et al. (2014)
Rye (*Secale cereale* L.)	Immature embryo	0.03	Popelka and Altpeter (2003)
Oats (*Avena sativa* L.)	Immature embryo	0.12	Gasparis et al. (2008)
Fox tail millet (*Setaria italica* L.)	Callus	0.06	Wang et al. (2011)
Finger millet (*Eleusine coracana* (L.) Gaertn.)	Callus	0.04	Antony Ceasar and Ignacimuthu (2011)
Pearl millet [*Pennisetum glaucum* (L.) R. Br.]	Callus	0.03	Ramadevi et al. (2014)
Sugar cane (*Saccharum officinarum* L.)	Pre-cultured seed	0.45	Mayavan et al. (2013)
Bermuda grass (*Cynodon dactylon* L.)	Callus	0.05	Salehi et al. (2005)
Bentgrass (*Agrostis stolonifera* L.)	Callus	0.03	Yu et al. (2000)
Italian Ryegrass (*Lolium multiflorum* Lam)	Callus	0.07	Lee et al. (2010)
Perennial Ryegrass (*Lolium perenne* L.)	Callus	0.2	Patel et al. (2013)
Tall fescue (*Festuca arundinacea* Schreb.)	Callus	0.08	Dong and Qu (2005)
*Zoysia japonica* Steud.	Stolon node	0.07	Ge et al. (2006)
Switch grass (*Panicum virgatum* L.)	Callus	1.0	Li and Qu (2011)
Chinese silvergrass (*Miscanthus sinensis Andersson*)	Callus	0.01	Hwang et al. (2014)
*Brachypodium distachyon* (L.)	Callus	0.67	Steinwand et al. (2013)

High end values found in the literature are displayed. *The explants were sectioned into pieces after the co-cultivation so that many transgenic events were recovered from a single original explant.

Many laboratories cannot afford such an investment, and most recent studies of transformation methods of rice still focus on calli, as exemplified as follows. Saika and Toki (2010) observed that calli of the indica cultivar Kasalath were transformed 10 times as efficiently as was the japonica cultivar Nipponbare. Karthikeyan et al. (2011) found that callus induced from the leaf bases of germinating seeds was good for transformation of indica cultivar ADT 43. Sahoo et al. (2011) optimized transformation protocols for seed-derived callus of four indica cultivars. Sahoo and Tuteja (2012) modified media composition for calli for indica IR64. Ozawa and Takaiwa (2010) observed a 5–10 fold increase in the frequency of transformation by using the calli from suspension cultures of Nipponbare. Duan et al. (2012) optimized a protocol for japonica Wanjing 97 and Nipponbare. The use of calli from mature seeds may be associated with a problem of genotype dependence, but it is quite convenient as long as the protocols for the cultivars of interest are in place.

Rice is also an excellent model plant for molecular biology and genomics, and the highly efficient transformation capability has been extensively exploited in the studies of various new technologies, such as gene targeting by homologous recombination followed by the elimination of a selectable marker gene from targeted loci without leaving footprints (Nishizawa-Yokoi et al., 2014), sequence-specific genome modification by CRISPR/Cas9 (Shan et al., 2013), excision of DNA by site-specific recombinases (Akbudak and Srivastava, 2011), elimination of selection marker genes (Komari et al., 1996), reduction of the transfer of so-called vector-backbone sequences (Kuraya et al., 2004), construction of T-DNA tagging libraries (Ryu et al., 2004) and high-throughput complementation of mutation by genes cloned by a map-based approach (Komori et al., 2004). Technologies tested first in rice are then expected to be applied to other cereals.

## PROGRESS IN MAIZE TRANSFORMATION

Maize is the premier crop of the agricultural biotechnology business. Maize is the only cereal crop that has genetically modified varieties on the market. Maize was the second cereal crop in the development of an efficient transformation protocol mediated by *A. tumefaciens*. Two years after the breakthrough in rice, Ishida et al. (1996) reported the production of transgenic maize from the immature embryos co-cultivated with *A. tumefaciens* in inbred A188. The protocols were well optimized in subsequent studies (Ishida et al., 2007), and the frequency of independent transformants reached 50% per explant infected (**Table 2**). Transgenic maize plants quickly entered the development pipelines of major biotechnology companies, and the transgenic maize events that were deregulated after 2003 are mostly progeny from the plants transformed with *A. tumefaciens* (International Service for the Acquisition of Agri-biotech Applications^4^). Genetically modified maize varieties, which carry traits such as insect resistance and herbicide tolerance, are now grown on 57.4 million acres globally.

Still, current transformation protocols in maize are inconvenient in at least two aspects. Firstly, while calli and other tissues are frequently employed in rice transformation, efficient transformation has been performed almost always using immature embryos in maize (Ishida et al., 2007). The quality of the immature embryos is very important in all cereals, but it is also very sensitive to environmental condition changes, and a good facility and a team of experts are needed. The size of the embryos is a good indicator of the developmental stage, which is critically important, and embryos that are between 1.2 and 1.5 mm in length along the axis are optimal for maize transformation.

Unlike rice, maize transformation is routinely carried out mostly by elite laboratories, such as ones of leading universities and multi-national seed/biotechnology companies. Fortunately, some of the major universities, such as Iowa State University^5^ and the University of Missouri^6^, have been offering maize transformation service to academic laboratories.

Secondly, genotype dependence of the transformation technology seems to be greater in maize than in rice. Inbred A188 is a genotype quite often studied in tissue culture and the first genotype transformed efficiently by *A. tumefaciens* (Ishida et al., 1996). Another genotype Hi-II, also popular in tissue culture, was subsequently transformed (Zhao et al., 2001; Frame et al., 2002). In other genotypes, transformation was possible only at a very low frequency or not at all. Both A188 and Hi-II display poor agronomic characteristics and may be used only in basic studies or in initial testing of trait genes. In the programs to produce genetically modified varieties, these genotypes may be initially transformed and then hybridized with elite varieties, or elite genotypes could somehow be transformed at a very low frequency in large scale experiments.

Expansion of the genotypes that can be transformed efficiently has been one of the most important objectives in maize. The efforts are on-going. Treatment of the immature embryos with heat and centrifugation before infection elevated the efficiency of transformation in several inbreds (Hiei et al., 2006). Frame et al. (2011) described modification of culture media to elevate the transformation frequency of inbred B104. Sato et al. (2011) reported the selection of transformants of Japanese inbred line Mi29 by the combination of mutant acetolactate synthase (ALS) and a herbicide, Bispyribac-sodium; transformants were obtained from 30% of the immature embryos infected. Akoyi et al. (2013) adjusted plant growth regulators in culture media for immature embryos from five tropical maize varieties. Ombori et al. (2013) examined factors, such as strains of *A. tumefaciens*, types of vectors and duration of co-cultivation of maize tissues and *A. tumefaciens*, in the transformation methods for six inbreds and two hybrids of tropical maize.

During the process of development of a commercial transgenic variety, 1000s of transformants are screened for numerous desired characteristics, such as single copy integration, absence of vector-backbone sequences and other unnecessary foreign DNA segments, adequate level and regulation of transgene expression, proper stacking of trait genes, absence of alteration of non-target traits, other aspects of high quality transformants, and good overall performance. Technologies to improve the efficiency of the process are highly desired, and quite a few approaches are currently being tested. Such technologies are especially needed in maize. For example, backbone-free, low-copy-transgene maize plants were generated by delivering T-DNA from the *picA* locus of the chromosome of *A. tumefaciens* (Oltmanns et al., 2010). Targeted mutagenesis was performed in maize by zinc-finger nuclease (Shukla et al., 2009), mega-nuclease (Gao et al., 2010) and by TALENs and CRISPR/Cas9 (Liang et al., 2014). New technologies tested in maize are still limited but will likely be expanded significantly in the near future.

## PROGRESS IN WHEAT TRANSFORMATION

Wheat is the number one crop in the world in many ways. It is the most favored food staple, and both the global acreage of wheat cultivation and the volume of wheat grain internationally traded are the largest among all the crops (**Figure 1**; OECD-FAO Agricultural Outlook 2014–2023^7^). However, wheat is far behind other major cereals in the research and application of biotechnology. It is not that scientists are not interested in wheat. On the contrary, wheat is one of the most extensively studied species in various disciplines in biology and agricultural sciences. The term “genome” was coined from the study of wheat, but, ironically, the complex hexaploid structure and the large size of the wheat genome have been among the technical hurdles in the study of this species.

The fact that wheat has been quite recalcitrant to tissue culture and genetic transformation has been a major hurdle that delayed biotechnology applications in this crop. Again, the start of the efforts to develop transformation methods in wheat was not belated. The first transgenic wheat was created by direct gene transfer in the early 1990s (Vasil et al., 1992). Soon after efficient protocols of transformation mediated by *A. tumefaciens* were developed in rice and maize, wheat, cultivar Bobwhite, was transformed by *A. tumefaciens* (Cheng et al., 1997) and quite a few related reports followed. As with other cereals, the choice of the starting material was immature embryos. However, progress thereafter made in wheat was slow, and the frequency of transformation was mostly less than 5% of the inoculated tissue pieces in later reports (Wan and Layton, 2006; Wu et al., 2008; Risacher et al., 2009; He et al., 2010; Bińka et al., 2012).

Recently, Ishida et al. (2014) found that cultivar Fielder was higher than Bobwhite in the frequency of transformation and optimized the protocol to obtain a frequency of transformation of 90% (**Table 2**). They noticed that the list of key factors in wheat transformation, including choice of genotype, quality and stage of immature embryos, media composition, strain of *A. tumefaciens*, pre-treatment of embryos and handling of tissues, was not much different from those studied in rice and maize but that the optimal ranges of many of the factors were very small in wheat, speculating that the narrow windows were a key reason for the slow progress. Transformability could be genotype dependent in wheat (Abid et al., 2014), although varietal differences in efficiency of transformation have not been studied extensively yet. Like rice and maize, the size of immature embryos is a good indicator for the developmental stage, but the optimal size, between 2.0 and 2.5 mm in length along the axis, in wheat was larger than that in rice and maize (Ishida et al., 2014). Centrifuging immature embryos before infection was a critical step in the protocol, but heat shock was not effective, unlike with other cereals (Ishida et al., 2014).

## PROGRESS IN BARLEY TRANSFORMATION

Barley is a major staple crop in relatively dry regions of the world because of remarkably strong drought tolerance. It is the fourth cereal in farming acreage (**Figure 1**). The importance of this crop for humans is as great as the three major cereal crops described above as a food and feedstock and for the production of beer and syrup, although the amount of production in the world is far below the level of the three major crops. In addition, knowledge gained from the studies of barley, which has a simple diploid genome, might be useful for the studies of wheat which has a complex hexaploid genome.

Transformation mediated by *A. tumefaciens* of barley cultivar Golden Promise was reported soon after the success in rice and maize transformation (Tingay et al., 1997). Initially, the frequency of transformants was not very high, between 1.7 and 7.0% of the immature embryos infected, but recent studies reported the frequency around 25% or higher (Bartlett et al., 2008; Hensel et al., 2008; Harwood, 2014). The highest frequency in the study by Hensel et al. (2008) was 86.7%. Genotype dependence was also observed by them. Nine other cultivars were tested, and three of them could not be transformed. The frequency for the other cultivars was somewhat lower than Golden Promise and ranged between 0.5 and 12%.

Therefore, transformation methods for barley have been well established by now and could be employed in basic and applied studies quite efficiently. The transformation process in barley may be less efficient than in rice, as efficient as in wheat, but more efficient than in maize and other cereals.

## PROGRESS IN SORGHUM TRANSFORMATION

Sorghum (*Sorghum bicolor* L.) is the number five cereal in terms of production area (**Figure 1**). This is the major staple crop in a number of Sub-Saharan countries. Sorghum is very important globally for feed and has potential for ethanol production because of its high biomass yield and good drought tolerance.

Zhao et al. (2000) were the first to report *Agrobacterium*-mediated transformation of sorghum. The frequency of transformation of 10.1% per immature embryo co-cultivated with *A. tumefaciens* was quite high as an early study compared to the examples in many other cereals. However, the frequency reported since then has remained below 10%, although a number of laboratories extensively tried to optimize media compositions, pre-treatment of the embryos and other factors (Gao et al., 2005; Gurel et al., 2009; Kumar et al., 2011). Cultivars P898012 and Tx430 were mainly studied by these groups; other varieties were also transformed, but at somewhat lower frequency. Recently, Wu et al. (2014) added copper sulfate and 6-benzylaminopurine to the media for culturing tissues after infection and recorded the frequency of 33.2% for Tx430. Thus, sorghum has finally joined the list of the cereals that can be transformed efficiently by *A. tumefaciens*.

## PROGRESS IN TRANSFORMATION OF OTHER CEREALS AND GRASSES

In addition to the five species described above, diverse species of cereals and grasses are grown by humans for food, industrial feedstock, turf, silage, hay, and other applications. Quite a few species may now be transformed by *A. tumefaciens* (**Table 2**). Some of them may not be considered as crops. For example, *Brachypodium*, which is famous for its small genome size, and *Setaria*, which is considered as a model plant for C4 crops, have been studied mainly for research purposes. Switch grass and *Miscanthus* could be important bio-mass crops for fuel production in the near future.

The frequency of transformation of the species other than the top five cereals was generally low, probably because calli have been used as the main target tissues. The seeds/grains of plants that are not major food crops are usually tiny, and immature embryos may be too small to be conveniently collected and handled. An alternative is to induce calli from whatever tissues that are available for infection with *A. tumefaciens*, but calli often show much lower transformation frequency than immature embryos do.

Therefore, transformation of the species listed in **Table 2** other than the top five cereals needs improvement. More efforts in tissue culture and transformation are needed if these species are to become routine study materials by molecular biologists.

## CONCLUSION

The progress made in cereal transformation during the last two decades is remarkable. The top five cereals are now quite efficiently transformed by *A. tumefaciens*. Technology for the major cereals has reached the point where specific genes may be tested in sufficient numbers of transgenic events in basic and applied studies. Quite a few other cereal and grass species may also be transformed by *A. tumefaciens*, and, although the efficiency of transformation is currently not high, they are at least ready for optimization studies if necessary. Learning on successes achieved in the major cereals may also be applicable to other cereals.

Of course, there are still hurdles. The genotype dependence is a major one. Many varieties of rice may be transformed, but, in other cereals, only limited genotypes are efficiently transformed. Thus, the expansion of the number of genotypes that can be efficiently transformed is a key task for cereals. This task is not easy because the reasons for or mechanisms behind the varietal differences are poorly understood. A rare example was a finding that low nitrate reductase activity caused a poor tissue culture response in certain rice cultivars, and this finding led to development of improved tissue culture protocols and transformation of these cultivars (Ozawa and Kawahigashi, 2006).

Rice could play a pivotal role as a model to develop new transformation protocols. Many genotypes of rice can be transformed at a frequency at least an order of magnitude higher than other cereals. The advantage of rice is in the small genome size and in the small plant size compared to maize and sorghum. Therefore, it is a convenient approach to test genes first in rice and then in target cereals.

Dependence on immature embryos is another issue that needs to be addressed. Immature embryos are definitely the best target tissue for cereal transformation, but the quality of the embryos is greatly affected by vegetative conditions of the mother plants. Therefore, cereal transformation can be efficiently conducted only by an elite team with a suitable facility and expert staff.

On the other hand, calli are a convenient target tissue, which could be supplied year-round without special facilities, if an efficient protocol for the genotype of interest is in place. Therefore, it is understandable that many laboratories are making efforts to culture calli of genotypes of interest in rice and of other cereals. However, collaboration with elite transformation teams, who are well resourced and can handle immature embryos of the genotypes, would likely be much more productive than such efforts and are thus highly recommended.

It is true that we are far from an ideal world, where a single copy of a given piece of DNA, which could be very large, can be integrated into a given location of the genome of a given genotype of a given cereal species in a highly efficient manner without any rearrangements, any unnecessary DNA or any effects on non-target traits at a given time of the year with a minimum labor, but we can say we are making a good progress in the right track.

## Conflict of Interest Statement

The authors declare that the research was conducted in the absence of any commercial or financial relationships that could be construed as a potential conflict of interest.

## Footnotes

1 http://apps.fas.usda.gov/psdonline/circulars/production.pdf

2 http://www.un.org/en/development/desa/population/theme/trends/index.shtml

3 http://www.ipcc.ch/report/ar5/wg2/

4 http://www.isaaa.org/

5 http://agron-www.agron.iastate.edu/ptf/

6 http://plantsci.missouri.edu/muptcf/services/maize.html

7 http://www.oecd.org/site/oecd-faoagriculturaloutlook/
